# Generation of advanced fire blight-resistant apple (*Malus* × *domestica*) selections of the fifth generation within 7 years of applying the early flowering approach

**DOI:** 10.1007/s00425-018-2876-z

**Published:** 2018-03-14

**Authors:** Ina Schlathölter, Melanie Jänsch, Henryk Flachowsky, Giovanni Antonio Lodovico Broggini, Magda-Viola Hanke, Andrea Patocchi

**Affiliations:** 1 Agroscope, Research Division Plant Breeding, Schloss 1, P.B, 8820 Wädenswil, Switzerland; 2 Julius Kühn-Institut, Institute for Breeding Research on Fruit Crops, 01326 Dresden, Germany; 3 Present Address: Swiss Federal Institute of Technology, Molecular Plant Breeding, 8092 Zurich, Switzerland

**Keywords:** *BpMADS4*, *Erwinia amylovora*, Juvenility, Null segregant, Rapid crop cycle breeding

## Abstract

The approach presented here can be applied to reduce the time needed to introduce traits from wild apples into null segregant advanced selections by one-fourth.

Interesting traits like resistances to pathogens are often found within the wild apple gene pool. However, the long juvenile phase of apple seedlings hampers the rapid introduction of these traits into new cultivars. The rapid crop cycle breeding approach used in this paper is based on the overexpression of the birch (*Betula pendula*) *MADS4* transcription factor in apple. Using the early flowering line T1190 and ‘Evereste’ as source of the fire blight resistance (*Fb_E* locus), we successfully established 18 advanced selections of the fifth generation in the greenhouse within 7 years. Fifteen individuals showed the habitus expected of a regular apple seedling, while three showed very short internodes. The null segregants possessing a regular habitus maintained the high level of fire blight resistance typical for ‘Evereste’. Using SSR markers, we estimated the percentage of genetic drag from ‘Evereste’ still associated with *Fb_E* on linkage group 12 (LG12). Eight out of the 18 selections had only 4% of ‘Evereste’ genome left. Since genotypes carrying the apple scab resistance gene *Rvi6* and the fire blight resistance QTL *Fb_F7* were used as parents in the course of the experiments, these resistances were also identified in some of the null segregants. One seedling is particularly interesting as, beside *Fb_E*, it also carries *Fb_F7* heterozygously and *Rvi6* homozygously. If null segregants obtained using this method will be considered as not genetically modified in Europe, as is already the case in the USA, this genotype could be a very promising parent for breeding new fire blight and scab-resistant apple cultivars in European apple breeding programs.

**Electronic supplementary material:**

The online version of this article (10.1007/s00425-018-2876-z) contains supplementary material, which is available to authorized users.

## Introduction

Apple production requires the second highest pesticide application per hectare (Eurostat 2002). The treatment frequency index of apple in 2011, 2012, and 2013 in Germany, for instance, was about 32. This means that 32 pesticides were applied each season at maximum approved quantity. More than 80% of these pesticides were fungicides used to control fungal diseases such as apple scab and powdery mildew (Rossberg and Harzer 2015). The use of resistant or less susceptible cultivars would reduce the application of chemicals in apple production. Depending on the pathogen or pest, sources of resistance can be found in commercial or heritage cultivars, or in wild accessions of different *Malus* species. If resistance is present in commercial or heritage cultivars, then the F1 generation of resistant genotypes will already possess characteristics desired by the market. However, this is highly improbable if the source is introduced from a wild apple accession. In this second scenario, a cross with the “wild” resistant genotype is first required. Subsequently, up to four additional crosses with high-quality apple genotypes are necessary to remove most of the undesired characteristics of the “wild” origin, which were introduced together with the trait of interest. In general, the juvenile phase of apple lasts 4–5 years but could also last as long as 12 years (Hanke et al. 2007). Hence, the introgression of traits from wild species could take more than 20 years. In the past, several agro-technical approaches have been developed attempting to reduce the juvenile phase of apple (see Le Roux et al. 2012 for a short summary); however, none of them have reduced this phase below 18 months (Hanke et al. 2007).

The long juvenile phase also poses a challenge for breeding in other fruits. For example, almond (*Prunus dulcis*), cherry (*Prunus avium*), and peach (*Prunus persica*) trees, all require 3–5 years juvenility, while citrus (*Citrus* spp.) trees require 5–10 years (McGarry et al. 2017), and plum (*Prunus domestica*) trees require 3–7 years (Srinivasan et al. 2012). Furthermore, the long-lasting juvenile phase slows down breeding of fast-growing hardwood trees like poplar (*Populus* spp., juvenility of 7–10 years, Hsu et al. 2006) and eucalyptus (*Eucalyptus* spp., juvenility of two to more than 10 years, McGarry et al. 2017), which are of interest for pulp, energy, and timber.

Transgenic approaches have been found to overcome juvenility in several crops. Overexpression of the *Arabidopsis FLOWERING LOCUS T* (*FT*) or *FT* homologs in *Malus* (Kotoda et al. 2010), *Citrus* (Endo et al. 2005), poplar (Hsu et al. 2006), plum (Srinivasan et al. 2012), and eucalyptus (Klocko et al. 2016), led to early flowering by reducing the juvenile periods of these species. Similarly, the loss of function of *TERMINAL FLOWER1* (*TFL1*), which is an inhibitor of *FT*, in apple (Kotoda et al. 2006) and pear (Freiman et al. 2012), also led to early flowering. In apple, a system was also developed to activate early flowering “on demand” by controlling the transcription of *FT* genes from poplar (*Populus trichocarpa*) with the heat-inducible soybean (*Glycine max*) promoter Gmhsp 17.5-E (Wenzel et al. 2013).

In *Citrus*, the overexpression of other flower-inducing genes like *LEAFY* (*LFY*) and *APETALA1* (*AP1*) from *Arabidopsis* also led to a reduction of juvenility (Peña et al. 2001). However, the transfer and overexpression of the same genes in apple did not induce early flowering (Zhu et al. 2009; Flachowsky et al. 2010).

As an alternative approach to the stable transformations described above, viral vectors can be used to deliver *FT* orthologs and induce flowering (Virus-Induced Flowering, VIF). This is possible, as the *FT* gene product is phloem-mobile and viruses diffuse through the phloem into the plant (McGarry et al. 2017). This approach was first shown in melon (*Cucurbita moschata*, Lin et al. 2007). VIF has been successfully applied in apple by an ectopic expression of *Arabidopsis FT* delivered by the *Apple Latent Spherical Virus* (ALSV) by Yamagishi et al. (2011). In another experiment, early flowering in apple was obtained using an ALSV vector that simultaneously promotes expression of the *Arabidopsis FT* gene while silencing the apple *MdTFL1* gene (Yamagishi et al. 2014). The advantage of VIF over the approaches using stable transformation is that viruses do not integrate into the genome of the hosts and most of them do not pass efficiently through the germline, hence virus-free seeds could be obtained (Johansen et al. 1994).

In apple, early flowering was also obtained by overexpressing the *FRUITFULL*-homolog *BpMADS4* of silver birch (*Betula pendula* Roth.) in the apple cv. ‘Pinova’ (Flachowsky et al. 2007a). Based on the early flowering line T1190, Flachowsky et al. (2009) provided the proof of concept that the approach can be used to reduce apple juvenility to a few months after seed planting. In the same year, using the same transgenic line, Le Roux et al. (2012) initiated a case study aiming at the development of fifth generation advanced selections carrying the highly efficacious fire blight resistance gene *Fb_E* from the ornamental apple cultivar ‘Evereste’. *Fb_E* has been mapped to the bottom of linkage group 12 (Durel et al. 2009) and tightly linked markers are available following a map-based cloning project (Parravicini et al. 2011). Le Roux et al. (2012) reported the generation and analysis of the first two generations of this case study. Reciprocal crosses between the line T1190 and ‘Evereste’ were performed in 2009 and seedlings carrying the genes *BpMADS4* and *Fb_E* were identified. These genotypes started flowering as early as 15 weeks after seed planting and their pollen was used to pollinate potted apple trees to generate plants of the BC’1 generation. BC’1 seedlings carrying *Fb_E* maintained the expected level of resistance to fire blight. The percentage of the remaining ‘Evereste’ genome in the BC’1 plantlets combining *BpMADS4* and *Fb_E* was estimated with a genome-covering set of single sequence repeat (SSR) markers. Two genotypes, BC’1_16 and BC’1_19, were identified having only about 14% remnants of the ‘Evereste’ background. This value is well below the expected average of 25% for plants of the second generation. The current status of the use of transgenic early flowering approaches in perennial crops has recently been reviewed (Callahan et al. 2016).

In this paper, we report the successful development of null segregants of the fifth generation (BC’4) carrying the *Fb_E* fire blight resistance gene within 7 years. The genotypes maintained a high level of resistance to fire blight. In addition, genotypes lacking the early flowering transgene *BpMADS4* recovered the habitus expected of a non-transgenic apple seedling. The implications of these results for apple breeding are discussed.

## Materials and methods

### Plant material

The development of advanced apple (*Malus* × *domestica* Borkh.) selections carrying the fire blight resistance of ‘Evereste’ *Fb_E* (Durel et al. 2009) using the early flowering line T1190 (Flachowsky et al. 2007a, 2009, 2011) started in 2009 (Le Roux et al. 2012). Le Roux et al. (2012) produced plants of the BC’1 generation and used these as parents to develop the BC’2 generation. The work presented here is based on this material.

### Growing conditions

Growing conditions were set according to Le Roux et al. (2012) with the following modifications: trees were grown under long-day conditions in summer (14 h light and 10 h without additional light) at 20 °C during the day and 18 °C at night. Relative humidity was maintained at 60% and the plants were watered automatically. From the beginning of December to end of February, the trees (without leaves) were maintained in the greenhouse cabin without additional heating (only anti-frost heating) and no additional light or humidity regulation.

Plants were grown in 3-L pots filled with Floradur^®^ Pot Cyclamen/Poinsettia substrate (Floragard Vertriebs-GmbH, Oldenburg, Germany) supplemented with the fertilizer Osmocote (Hauert HBG Dünger AG, Grossaffoltern, Switzerland). A sulfur vaporizer was utilized for powdery mildew control and the biological product Solbac (Andermatt Biocontrol AG, Grossdietwil, Switzerland) was used against fungus gnats larvae. The insecticides Movento Arbo/Movento SC (name changed in 2017; Bayer AG Crop Science, Zollikofen, Switzerland), Vertimec (Syngenta Agro AG, Dielsdorf, Switzerland) or Plenum (Syngenta) were used against aphids, spider mites, thrips, or white flies.

All experiments with flowering plants carrying the flowering gene *BpMADS4* were performed in biosafety level 2 greenhouses. The first crosses between T1190 and ‘Evereste’ were done at the Swiss Federal Institute of Technology (Zürich, Switzerland), the subsequent ones were done at the Federal Research Station Agroscope (Wädenswil, Switzerland).

### Foreground selection of seedlings

DNA was extracted using the Extract-N-Amp™ Plant PCR kit (Sigma-Aldrich^®^, St. Louis, MO, USA). Presence of the *BpMADS4* transgene and the *nptII* (neomycin phosphotransferase II) gene was investigated according to Flachowsky et al. (2007a). As internal PCR control, RUBISCO (ribulose-1,5-bisphosphate carboxylase/oxygenase activase) primers (Flachowsky et al. 2007b) were multiplexed to the *BpMADS4* and *nptII* primers.

Presence of the *Fb_E* locus was assessed using the SSR markers ChFbE01 (GA19), ChFbE09, ChFbE02, and ChFbE06 (TATris16) (Parravicini et al. 2011). SSR markers were labeled with HEX, FAM or according to Schuelke (2000) as described by Parravicini et al. (2011). Only plants amplifying all four favorable alleles 266, 249, 230, and 273 bp of the SSR markers ChFbE01, ChFbE09, ChFbE02, and ChFbE06, respectively, were considered as carrying *Fb_E*.

### Estimation of the percentage of “Evereste’ genome on linkage group 12

Six linkage group 12 (LG12) SSR markers: CH05d04, CH04g04, Ch04d02, CH01f02, CH03c02, and Hi07f01 (Liebhard et al. 2002; Silfverberg-Dilworth et al. 2006), were used to estimate the percentage of ‘Evereste’ genome of the seedlings of the fifth generation carrying the *FB_E* locus, but not the *BpMADS4* cassette. Positions of the SSRs in Centimorgan (cM) were determined using the ‘Discovery’ LG 12 genetic map of Silfverberg-Dilworth et al. (2006) (rounded values). These are 13 cM for CH05d04, 29 cM for CH04g04, 51 cM for CH04d02, 56 cM for CH01f02, 64 cM for CH03c02, and 76 cM for Hi07f01. The position of the *Fb_E* locus is about 7 cM distal from Hi07f01 (Parravicini 2010) and for this estimation considered to be at the end of the LG. Total length of the LG 12 is therefore 83 cM. When a recombination event between flanking markers was identified, it was assumed to occur in the middle of the interval delimited by the two markers.

### Fire blight resistance assessment

Fire blight resistance of the 15 genotypes of the last generation with a normal habitus and carrying the *Fb_E* locus, but not *BpMADS4*, was assessed. Twelve replicates per genotype were grafted on M9 rootstocks and grown in a greenhouse. Only shoots equal or longer than 12 cm were inoculated. Inoculation using the *E. amylovora* strain CFBP 1430 at a concentration of 1 × 10^9^ cfu/ml was done according to Khan et al. (2007).

### Verification of the presence of *Rvi6* and *Fb_F7* in the 5th generation

The presence of *Fb_F7* in the 18 genotypes of the last generation was assessed using the SCAR markers AE10-375 and GE-8019 according to Khan et al. (2007). Similarly, the presence of *Rvi6* was assessed using the SSR marker CH-Vf1 and the SCAR marker AL07 according to Vinatzer et al. (2004) and Frey et al. (2004), respectively.

## Results

### Introgression of *Fb_E* in advanced apple selections

The introgression of *Fb_E* started in 2009 when reciprocal crosses between ‘Evereste’, the source of the fire blight resistance gene, and the early flowering line T1190 were performed. Le Roux et al. (2012) presented the development of the first two generations. For easy understanding, their development is shortly summarized in the following, with statistics included in Suppl. Table S1. In 2010, 19 out of 62 seedlings (30%) carried *Fb_E* combined with *BpMADS4*. Three of them, TxE_71, TxE_84, and ExT_5 were used to generate BC’1 progeny plants. In 2011, nine of 25 available *Fb_E*/*BpMADS4*-seedlings flowered sufficiently early in the season (between 14 and 25 weeks after seed planting). These plants were pollinated with ‘Royal Gala’ pollen and, with the exception of BC’1_16, all produced fruits. At the end of the 2011 season, 15 apples were harvested and 82 seeds of the BC’2 generation were collected and stratified. In 2012, 12 *Fb_E*/*BpMADS4* out of 72 generated seedlings, started flowering between 19 and 28 weeks after seed planting, and produced 45 fruits. 111 seeds were collected from these apples which were derived from nine BC’2 plantlets. However, 12 apples of three BC’2 plantlets were seedless. In 2012, the seedling BC’1_16 that did not flower in 2011 but was estimated to carry about 14% of ‘Evereste’ genome (Le Roux et al. 2012), was propagated and three plantlets were pollinated with pollen of ‘Royal Gala’ or ‘Granny Smith’. 17 out of 81 generated BC’2 seedlings carried the combination *Fb_E*/*BpMADS4*.

In 2013, BC’3 and BC’2_2012 (derived from BC’1_16) *Fb_E*/*BpMADS4* seedlings started flowering between 8 and 30 weeks and between 9 and 13 weeks after seed planting, respectively. Three seedlings did not flower. The flowering seedlings were pollinated with ‘Braeburn’ pollen but the three BC’3 and 15 BC’2_2012 apples that developed, were seedless. The BC’3 and BC’2 *Fb_E*/*BpMADS4* seedlings that survived until 2014 were pollinated with pollen of four different cultivars ‘Fuji’, Modi^®^, ‘Ladina’ and Kanzi^®^. 42 and 19 apples containing 183 and 118 seeds were obtained from 12 BC’3 and four BC’2_2012 *Fb_E*/*BpMADS4* seedlings that produced fruits.

As the production of seeds in 2014 was quite prolific, not all available seeds were stratified, i.e., no seeds gained form BC’2_43_2012 pollinated with Kanzi^®^ and Modi^®^, no seeds from BC’2_82_2012 and only 20 seeds each gained from BC’3_46 and BC’3_68 were stratified. In 2015, seedlings of the fifth generation or of the fourth generation derived from BC’1_16 were obtained. Out of the 113 BC’4 and 50 BC’3_2014 seedlings (the latter derived from BC’1_16), 28 (24.7%) and 12 (24%) seedlings carrying only the *Fb_E* resistance locus were identified. Eighteen BC’3_2014/BC’4 seedlings could be established in the greenhouse. Their pedigree is shown in Table 1.

**Table 1:** Pedigree and fire blight resistance of the 18 BC’3_2014/BC’4 seedlings; percentage of remaining ‘Evereste’ genome on LG12; fire blight resistance levels and results of the verification of the presence of the apple scab resistance gene *Rvi6* and fire blight QTL *Fb_F7* verified by molecular markers

2009	2010	2011	2012	2014	2015	% Evereste on LG12^a^	PLL3	SD (%)	*n*	LSD *P *< 0.05	*Fb_F7* ^g^	*Rvi6* ^h^
T1190 × Evereste	‘Topaz’ × T × E_F1_81		BC’1_16 × RG	BC’2_22_2012 × Modi^®^	BC’3_2014_10^e^	52^b^	n.a.	–	–			*Rvi6rvi6*
T1190 × Evereste	‘Topaz’ × T × E_F1_81		BC’1_16 × RG	BC’2_43_2012 × Fuji	BC’3_2014_17	4^c^	0.0	0.0	5	d	–	*Rvi6rvi6*
T1190 × Evereste	‘Topaz’ × T × E_F1_81		BC’1_16 × RG	BC’2_43_2012 × Ladina	BC’3_2014_30	4^c^	0.0	0.0	12	d	–	*Rvi6rvi6*
T1190 × Evereste	‘Topaz’ × T × E_F1_81		BC’1_16 × RG	BC’2_43_2012 × Ladina	BC’3_2014_36	4^c^	3.4	5.8	9	cd	–	*Rvi6Rvi6*
T1190 × Evereste	‘Topaz’ × T × E_F1_81		BC’1_16 × RG	BC’2_43_2012 × Ladina	BC’3_2014_40	4^c^	4.0	6.3	9	cd	–	*Rvi6Rvi6*
T1190 × Evereste	‘Topaz’ × T × E_F1_81		BC’1_16 × RG	BC’2_43_2012 × Ladina	BC’3_2014_43	4^c^	1.8	3.1	12	cd	–	–
T1190 × Evereste	‘Topaz’ × T × E_F1_81		BC’1_16 × RG	BC’2_43_2012 × Ladina	BC’3_2014_47	4^c^	0.8	2.5	11	d	Yes	*Rvi6Rvi6*
T1190 × Evereste	Topaz’ × T × E_F1_81	BC’1_19 × RG	BC’2_32 × GS^f^	BC’3_5 × Modi^®^	BC’4_6	16^d^	2.1	4.2	10	cd	–	*Rvi6rvi6*
T1190 × Evereste	Topaz’ × T × E_F1_81	BC’1_19 × RG	BC’2_32 × GS^f^	BC’3_5 × Modi^®^	BC’4_9	16^d^	0.0	0.0	10	d	Yes	*Rvi6rvi6*
T1190 × Evereste	Topaz’ × T × E_F1_81	BC’1_19 × RG	BC’2_32 × GS^f^	BC’3_5 × Modi^®^	BC’4_10^e^	4^c^	n.a.	–	–		–	–
T1190 × Evereste	Topaz’ × T × E_F1_81	BC’1_19 × RG	BC’2_32 × GS^f^	BC’3_5 × Modi^®^	BC’4_11^e^	16^d^	n.a.	–	–		Yes	–
T1190 × Evereste	Maloni Sally^®^ × T × E_F1_74	BC’1_7 × RG	BC’2_19 × GS	BC’3_68 × Kanzi^®^	BC’4_20	52^b^	6.1	6.9	8	bc	–	–
T1190 × Evereste	Maloni Sally^®^ × T × E_F1_74	BC’1_7 × RG	BC’2_19 × GS	BC’3_68 × Kanzi^®^	BC’4_21	52^b^	8.8	4.2	10	b	–	–
T1190 × Evereste	Maloni Sally^®^ × T × E_F1_74	BC’1_7 × RG	BC’2_20 × GS	BC’3_71 × Fuji	BC’4_46	4^c^	1.7	3.3	11	cd	–	–
T1190 × Evereste	Maloni Sally^®^ × T × E_F1_74	BC’1_7 × RG	BC’2_2 × GS	BC’3_39 × Modi^®^	BC’4_56	52^b^	0.8	1.5	9	d	–	–
T1190 × Evereste	Maloni Sally^®^ × T × E_F1_74	BC’1_7 × RG	BC’2_2 × GS	BC’3_46 × Kanzi^®^	BC’4_79	52^b^	3.2	3.4	12	cd	–	–
T1190 × Evereste	Maloni Sally^®^ × T × E_F1_74	BC’1_7 × RG	BC’2_2 × GS	BC’3_46 × Kanzi^®^	BC’4_81	52^b^	0.2	0.8	12	d	–	*Rvi6rvi6*
T1190 × Evereste	Maloni Sally^**®**^ × T × E_F1_74	BC’1_7 × RG	BC’2_2 × GS	BC’3_46 × Kanzi^®^	BC’4_82	52^b^	0.9	2.1	12	d	–	*Rvi6rvi6*
						Average BC’3_2014 and BC‘4 s	2.2	4.1				
						Evereste ‘13/‘14/‘16^i^	2.6	2.9	16	cd		
						Gala ‘13/‘14/‘16^i^	88.2	11.9	21	a		

*RG* Royal Gala, *GS* Granny Smith

^a^Based on six SSR markers spread over LG12 (CH05d04, CH04g04, CH04d02, CH01f02, CH03c02, Hi07f01), the genetic distances between the SSRs markers according Discovery 12 (Silfverberg-Dilworth et al. 2006) and the position of *Fb_E* estimated at the end of the LG at 7 cM from Hi07f01

^b^Recombination observed between CH04g04 and CH04d02

^c^Recombination between Hi07f01 and the block of four SSRs spanning the *Fb_E* locus

^d^Recombination observed between CH03c02 and Hi07f01

^e^“Ananas” phenotype, see text

^f^The correct cross is probably BC’2_32 × BC’1_16, see “Discussion”

^g^This fire blight resistance QTL is present in the cultivar ‘Ladina’ and ‘Topaz’

^h^This apple scab resistance gene is present in the cultivars ‘Evereste’, ‘Topaz’ and ‘Ladina’. *Rvi6Rvi6* and *Rvi6rvi6* indicate the presence of allele of *Rvi6* inducing resistance at the homo- and heterozygous state, respectively. “–” indicates the absence of the allele associated to resistance

^i^PLL3 average of the inoculation results of the years 2013, 2014 and 2016

In general, from F1 to BC’4, between 29 and 64 apples were harvested per generation, between 137 and 229 seeds were collected, between 62 and 153 seedlings were obtained, from which between 19 and 38 carried *Fb_E* and *BpMADS4* together, while the average time the seedlings of the F1 to the BC’3 generations needed to flower for the first time ranged from 18 to 27 weeks (Suppl. Table S1).

### Morphological observations

**Fig. 1:**
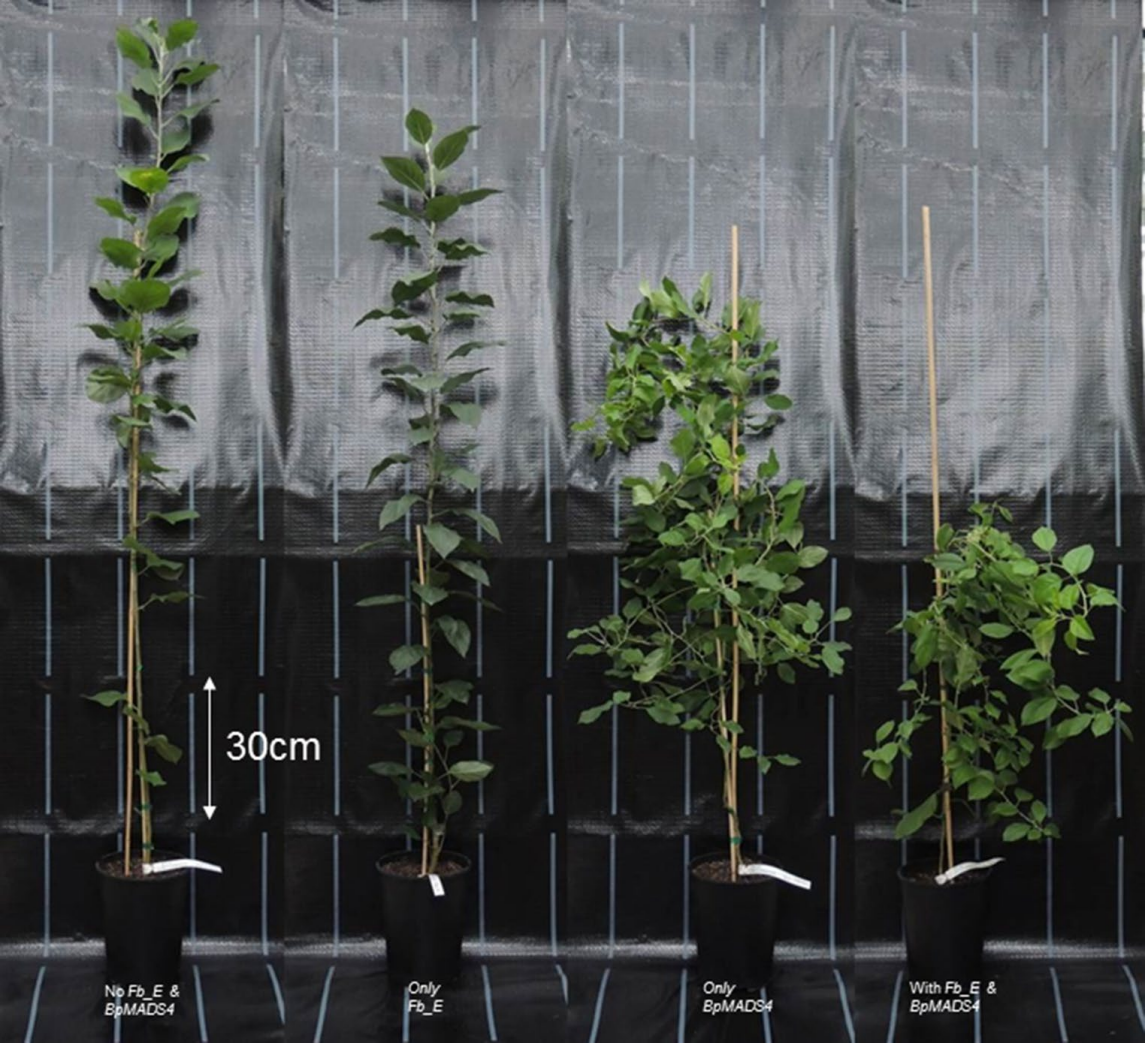
Habitus of four seedlings derived from the cross of BC’2_2 (*Fb_E/BpMADS4*) and ‘Granny Smith’, representing the four groups of seedlings (see picture) that are generated in each generation (N.B. *Fb_E* and *BpMADS4* are independently inherited). *BpMADS4* genotypes show the typical slender habitus. The seedlings without *BpMADS4* recover the habitus of a non-transgenic apple seedling

**Fig. 2:**
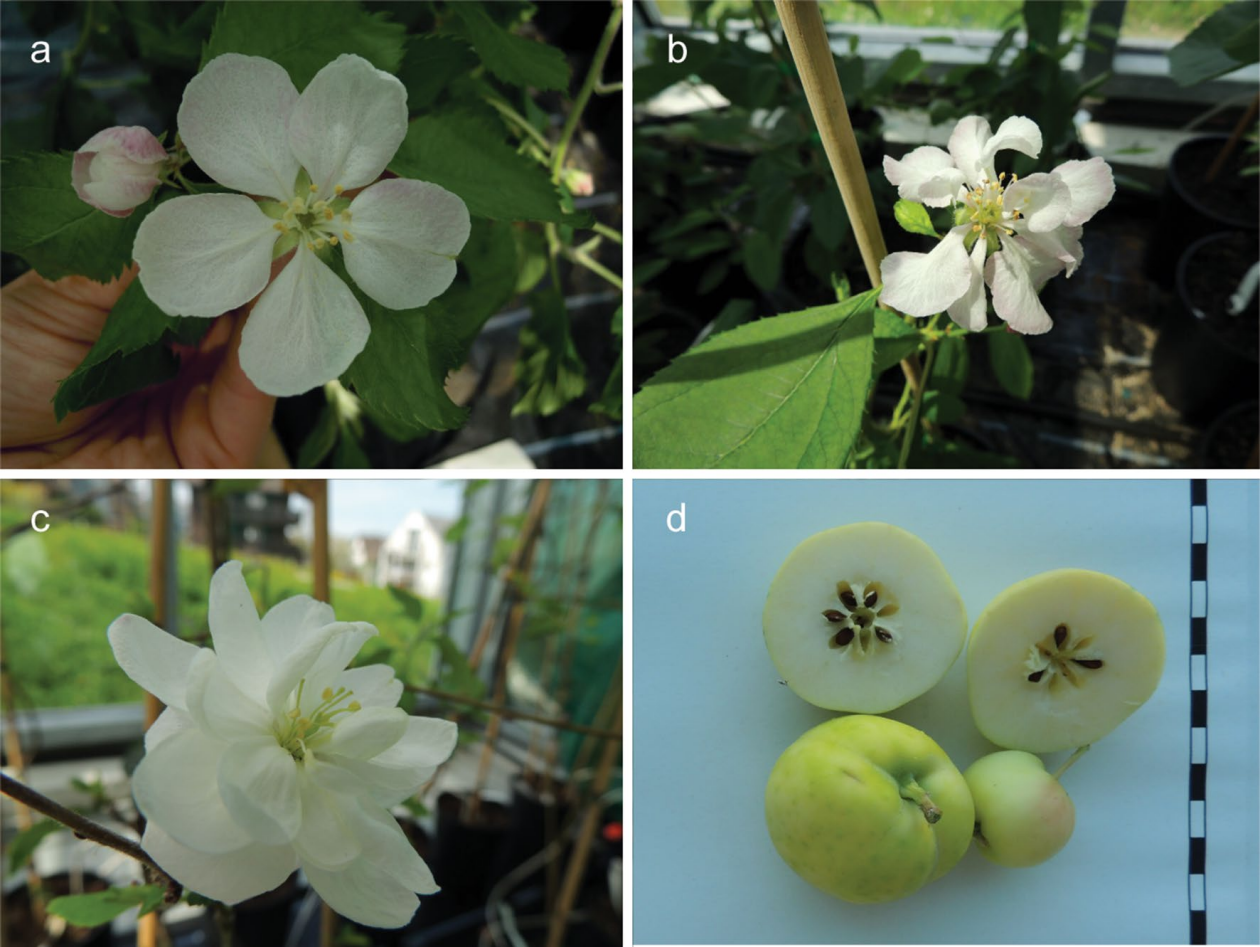
Pictures of transgenic flowers and fruit with abnormalities. **a** Flower without pistil. **b**, **c** Flowers with more than the expected five petals. **d** Apple fruit with seven instead of the expected five seed chambers

*BpMADS4* seedlings, with or without *Fb_E*, showed a typical slender habitus (Fig. 1). The shoots of the plantlets were always thin. Once a flower or a bunch of flowers was formed at the end of the shoot, shoot growth stopped and often lateral shoots started developing. This process led to quite bushy plantlets with hanging shoots. Over the generations, *BpMADS4/Fb_E* genotypes that did not flower or that produced flowers without pistils were observed (Fig. 2a). As these flowers are useless for breeding, the corresponding seedlings were systematically discarded. We also observed flowers with more than five petals and an apple with seven seed chambers instead of five (Fig. 2b–d).

**Fig. 3:**
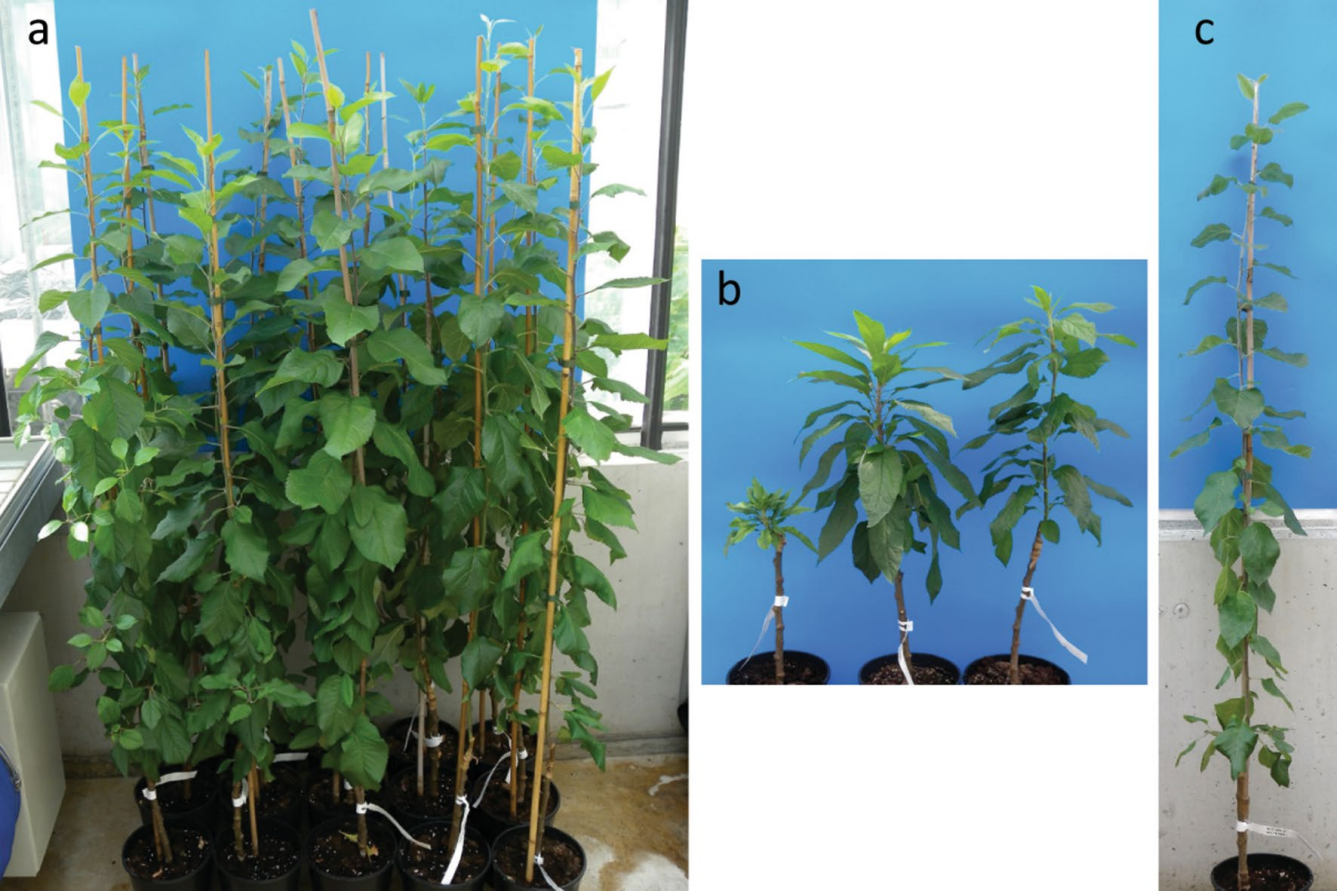
The 18 seedlings of the BC’3_2014/BC’4 generation carrying *Fb_E* and lacking *BpMADS4*. **a** 15 BC’3_2014/BC’4 seedlings showing a regular habitus for an apple seedling. **b** Three BC’3_2014/BC’4 seedlings showing a compact habitus we called ‘ananas’ (i.e., pineapple). **c** Seedling BC’3_2014_47

Seedlings that did not inherit the *BpMADS4* gene recovered the habitus expected for a normal non-transgenic young apple seedling. These plantlets have a main shoot and none or very few and relatively short lateral shoots (Fig. 1). This later habitus was observed for 15 out of the 18 BC’3_2014/BC’4 seedlings carrying only the *Fb_E* locus (Fig. 3a). On the contrary, the remaining three BC’3_2014/BC’4 (BC’3_2014_10, BC’4_10 and 11) showed a very compact habitus with a very short internode length and lanceolate leaves (Fig. 3b), which we named ‘ananas’ (i.e., pineapple).

The fruits of the BC’2_2012 and BC’3 seedlings that produced the 18 BC’3_2014/BC’4 seedlings were weighed, measured, and compared with the fruits of ‘Evereste’. The average fruit weight of these 40 fruits was 41.2 g (range 18.6–80.8 g) compared to 4.4 g (range 3.5–5.2 g) of those of ‘Evereste’, corresponding to a 9.4-fold weight increase. The heaviest fruit reached 117.6 g. The height and the width of the fruits ranged from 31.6 to 52.8 mm (average 39 mm) and 33.9 to 53.4 mm (average 42.4 mm) for the BC’2_2012 and BC’3 seedlings, respectively. The height and the width of ‘Evereste’ fruits ranged from 16 to 20.9 mm (average 18 mm) and 19.6 to 23.6 mm (average 21.1 mm), respectively. This corresponds to an approximately twofold increase in size on average for the fruits of the BC’2_2012 and BC’3 seedlings compared to ‘Evereste’, but relative increases of up to 2.9 times for height and 2.7 times for width have been observed (Table 2).

**Table 2:** Weight, size, and number of seeds of the 2014 fruits that generated the seedlings of the BC’3_2014/BC’4 generation

Genotype	Harvest	No. of fruits	Ø weight (g)	SD	Min	Max	Ø height (mm)	SD	Min	Max	Ø width (mm)	SD	Min	Max	Ø No. seeds	SD	Min	Max
Evereste	2015	20	4.4	0.5	3.5	5.2	18.0	1.3	16.0	20.9	21.1	0.9	19.6	23.6	4.1	1.0	2	6
BC’2_16_2012	2014	3	27.9	16.0	11.2	43.2	34.4	7.8	26.4	42.1	38.0	8.5	28.7	45.3	4.0	1.0	3	5
BC’2_22_2012	2014	2	22.8	–	20.7	24.9	31.6	–	31.6	31.6	36.7	–	35.3	38.1	6.5	–	4	9
BC’2_43_2012	2014	12	67.3	24.5	33.0	117.6	45.8	6.1	37.5	58.4	52.9	6.4	39.4	62.8	6.9	3.9	1	13
BC’3_5	2014	3	39.4	22.8	13.1	54.1	36.7	9.2	26.1	42.3	42.6	10.2	30.8	48.7	4.3	3.5	1	8
BC’3_68	2014	8	27.1	5.1	16.5	32.9	34.6	2.8	28.8	37.9	37.1	2.9	31.3	40.9	5.5	2.2	2	8
BC’3_71	2014	3	18.6	3.8	14.8	22.4	31.7	1.6	30.3	33.4	33.9	2.9	31.4	37.0	1.3	0.6	1	2
BC’3_39	2014	3	80.8	17.1	62.7	96.7	52.8	7.5	45.4	60.3	53.4	4.0	48.9	56.5	3.0	1.0	4	2
BC’3_46	2014	6	45.8	8.9	35.3	57.9	44.7	2.9	40.9	48.2	44.4	3.4	41.4	49.1	7.8	1.0	6	9
Average (BC’2_2012, BC’3)			41.2				39.0				42.4				4.9			
Increase relative to ‘Evereste’			9.4				2.2				2.0				1.2			
Max (BC’2_2012, BC’3)			80.8				52.8				53.4				7.8			
Max relative to ‘Evereste’			18.4				2.9				2.5				1.9			
Min (BC’2_2012, BC’3)			18.6				31.6				33.9				1.3			
Min relative to ‘Evereste’			4.3				1.8				1.6				0.3			

BC’2_2012 and BC’3 seedlings produced on average between 1.3 and 7.8 seeds, average 4.9 seeds, but up to 13 seeds were collected from a single apple. ‘Evereste’ produced between two and four seeds, average 4.1 seeds.

### Fire blight resistance

**Fig. 4:**
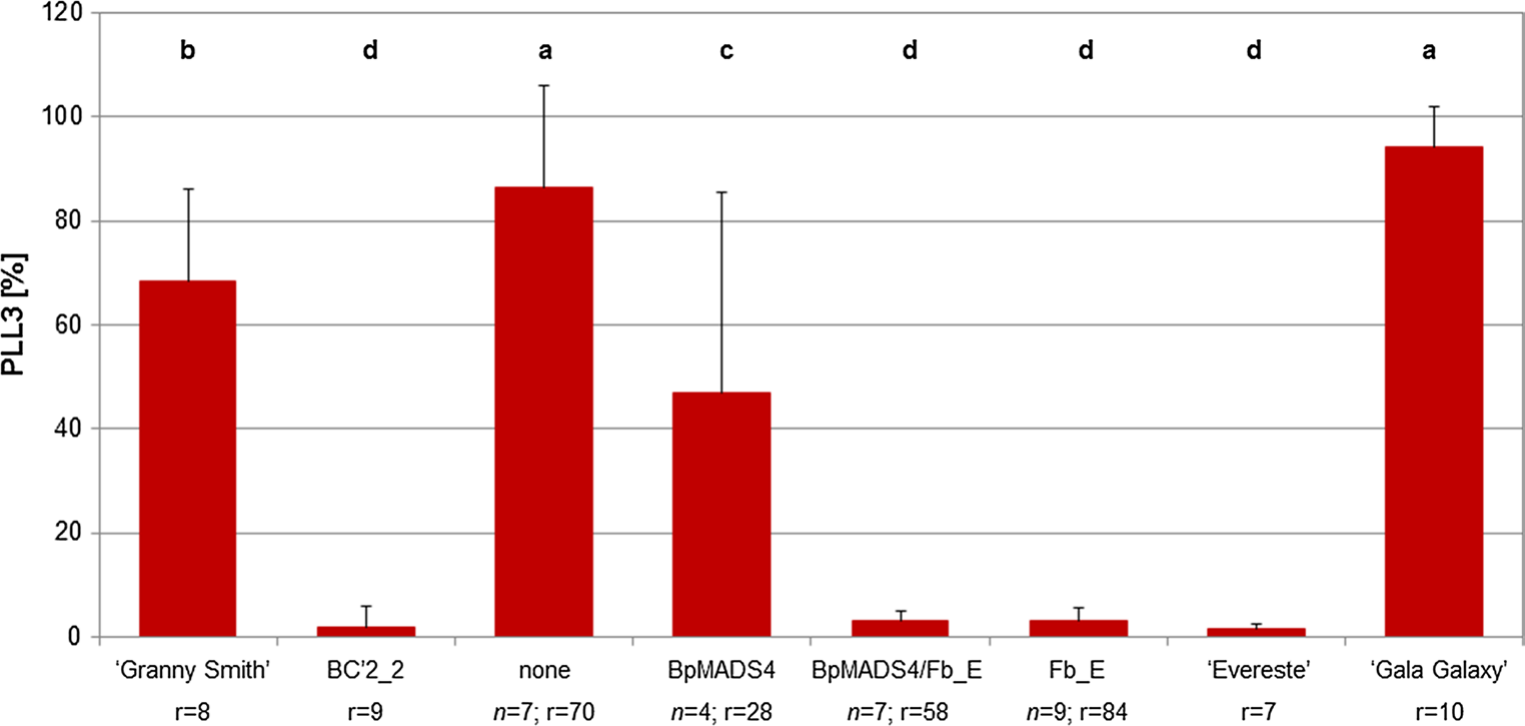
Fire blight resistance levels of BC’3 plants derived from the cross BC’2_2 (*Fb_E*/*BpMADS*4) and ‘Granny Smith’ subdivided into four groups depending on the inheritance of *Fb_E* and *BpMADS4*. The genotypes carrying *Fb_E* show an average resistance level similar to that of ‘Evereste’. *PLL3* percentage of shoot length showing fire blight symptoms after 3 weeks, *n* no. of genotypes, *r* no. of total replicates, *Gala* susceptible control, LSD *P* ≤ 0.05

Fire blight resistance was assessed for 27 BC’3 plants derived from the cross between BC’2_2 (*Fb_E*/*BpMADS*4) and ‘Granny Smith’, and subdivided in four groups depending on inheritance of *Fb_E* and *BpMADS4* (Fig. 4). The two groups carrying *Fb_E* with or without *BpMADS4* showed a resistance level similar to that of ‘Evereste’ on average. The group of genotypes that did not carry *Fb_E* and *BpMADS4* showed the lowest resistance level (similar to ‘Gala Galaxy’), while the group composed of genotypes carrying only *BpMADS4* showed an intermediate level of resistance.

The level of fire blight resistance of the six BC’3_2014 seedlings and nine BC’4 seedlings with a normal habitus carrying the *Fb_E* resistance was also assessed (Table 1). Their PLL3 values varied between 0 and 8.8 (average 2.2 ± 4.1). The susceptible control ‘Gala Galaxy’ showed a PLL3 of 88.2 ± 11.9 while the resistant control ‘Evereste’, showed a PLL3 of 2.6 ± 2.9. The PLL3 values of all BC’3_2014/BC’4 seedlings were not statistically different from ‘Evereste’ except for BC’4_21 (PLL3 8.8 ± 4.2) but all were statistically different from ‘Gala Galaxy’ (LSD *P *< 0.05).

### ‘Evereste’ LG12 background

The percentage remnant of ‘Evereste’ genome on LG12 was estimated for the 18 BC’3_2014/BC’4 seedlings (Table 1). Eight seedlings, BC’3_2014_17/30/36/40/43/47 and BC’4_10/46, showed recombination events in the interval between the SSR marker Hi07f01 and the four SSRs spanning the *Fb_E* locus. These recombination events took place while raising BC’2_43_2012, BC’2_20 and BC’4_10 and left about 4% of ‘Evereste’ LG12 in the seedlings (Table 1). These seedlings have the least genetic drag among the BC’3_2014/BC’4 seedlings. Three seedlings showed a recombination event between CH03c02 and Hi07f01 which occurred while raising BC’1_19. These seedlings have an estimated leftover of ‘Evereste’ LG12 of about 16%. The remaining seven genotypes showed a recombination event between the SSR markers CH04g04 and CH04d02 which occurred while rising BC’1_7 and BC’2_22_2012 and are still carrying 52% of the original ‘Evereste’ chromosome carrying the *Fb_E* locus.

### Presence of additional resistance QTLs and genes

Some of the parents used for crosses during the generation of the 18 BC’3_2014/BC’4 seedlings possessed the apple scab resistance gene *Rvi6* (‘Evereste’, ‘Topaz’, Modì^®^ and ‘Ladina’) as well as the major QTL for fire blight resistance *Fb_F7* (‘Ladina’ and ‘Topaz’). The presence of these resistance loci was assessed with molecular markers (Table 1). The *Rvi6* resistance gene was found in three seedlings at the homozygous (*Rvi6Rvi6*) and in seven at the heterozygous (*Rvi6rvi6*) state. *Fb_F7* was identified in three seedlings. Two of them carried both *Fb_F7* and *Rvi6*: BC’4_9 carries *Rvi6* at the heterozygous state, BC’3_2014_47 carries *Rvi6* at the homozygous state.

## Discussion

### The method and suggestions to improve it

The reduction of juvenility by overexpressing *BpMADS4* in the transgenic apple line T1190 described here is effective and could be efficiently used to develop advanced selections carrying genes of wild apple species. In this proof of concept experiment, two seedlings flowered as early as 8 weeks after seed planting, while the average flowering time of F1 to BC’3 seedlings ranged from 18 to 27 weeks, or 23 weeks if the three genotypes that took more than a year to flower are excluded. In fact, while aiming for rapid progress through the generations, such genotypes should be discarded.

Applying this method, we were able to produce seedlings of the 5^th^ generation 3–4 times faster than would have been possible by classical breeding (20–25 years). Indeed, the seedlings were obtained within 7 years from the first cross. Without the setback of apple production in 2013, the same result could have been obtained 1 year earlier. Verification of the vitality and germination rate of the pollen (Flachowsky et al. 2007a) used to pollinate the flowers of the *BpMADS4/Fb_E* seedlings was not included in our protocol. This confirmation would likely have indicated poor vitality and/or germination for the ‘Braeburn’ pollen we used in 2013, which we hypothesize to be the cause of the lack of seed production that year. Therefore, we recommend taking the extra step to perform the vitality and germination rate test, and to maintain a good stock of frozen pollen of different cultivars.

To verify the progress of the reduction of the ‘Evereste’ genome over the generations, we first estimated the percentage of ‘Evereste’ genome in the *BpMADS4/Fb_E* BC’1 seedlings with a set of SSR markers spanning the genome (Le Roux et al. 2012). The hypothesis was that seedlings that loose ‘Evereste’ alleles would be better candidates than those still having them. Instead of using only SSR markers, such a test can now be done even more efficiently using high-throughput genotyping methods like Genotyping By Sequencing (GBS) or apple single nucleotide polymorphism (SNP) arrays spanning the whole genome (Chagné et al. 2012; Bianco et al. 2014, 2016), as well as building haploblocks (Di Pierro et al. 2016) to generate more informative markers. Another strategy for prioritizing seedlings could be the application of Genomic Selection (GS), which has been reported in apple (Kumar et al. 2012a, b; Muranty et al. 2015). Application of GS for fruit and tree characteristics would be very useful for the identification of the best seedlings of each generation, but in particular for the final one. In fact, seedlings from F1 to BC’3 must be selected according to the duration they take to bloom. However, seedlings that flower very early, are not automatically the best ones, as very young seedlings are generally too small to support fruits until harvest, while seedlings that flower too late will delay the process. We observed that seedlings that started to flower between 14 and 28 weeks after seed planting produced apples (Suppl. Table S1, years 2010–2012, excluding ExT_F1_5 which did not flower in the same year as seed planting; 2013 no apples with seeds were produced and the seedlings pollinated in 2014 were all over 1 year old). Therefore, prioritization of *BpMADS4/Fb_E* seedlings by GS or other methods needs to be carefully thought out concerning blooming. As we had a relatively low number of *BpMADS4/Fb_E* seedlings per generation (between 19 and 38, Suppl. Table S1), we decided not to prioritize these seedlings according to the percentage of ‘Evereste’ leftover in their genome, but to work with all the seedlings that were flowering by the end of July, in order to harvest fruits by the end of the year at the latest. The exception was the seedling BC’1_16. BC’1_16 (as well as BC’_19) was found by Le Roux et al. (2012) to carry only about 14% of the ‘Evereste’ genome, which is expected for the average of the BC’2 generation. Hence, although BC’1_16 did not produce apples in 2011, the seedling was not discarded, but was propagated in 2012, and the flowers of these plantlets were pollinated with pollen from two different cultivars (Suppl. Table S1).

Parents carrying the apple scab resistance gene *Rvi6* (‘Evereste’, ‘Topaz’, Modì^®^, and ‘Ladina’) and the fire blight resistance QTL *Fb_F7* (‘Ladina’ and ‘Topaz’) were used in the course of the experiment. We did not select for these resistances over the generations, but the presence of these resistances in the final products has been molecularly tested (Table 1). As expected by the relatively high number of parents carrying *Rvi6*, seedlings carrying this resistance gene (homozygously or heterozygously) were found in 10 out of 18 BC’3_2014/BC’4 seedlings. The presence of *Rvi6* is noteworthy in BC’4_81 and BC’4_82. As in the pedigree of these two seedlings, only ‘Evereste’ carries *Rvi6*; therefore, this gene was not outcrossed over five generations (probability of 3.1%). Considering that no selection for apple scab was applied and that in the greenhouse no (obvious) selective advantage of being apple scab resistant can be expected, this can be ascribed to a chance or to an unknown factor tightly associated with *Rvi6* that gives a positive selective advantage under the conditions used in this experiment. Under “regular” breeding conditions, no segregation skewedness in favor of *Rvi6* carrying seedlings is observed (Baumgartner et al. 2015). One seedling, BC’3_2014_47 (Fig. 3c), looks particularly interesting. It carries *Rvi6* at the homozygous state, as well as *Fb_F7*, and is highly resistant to fire blight. It is also important to note that it is one of the genotypes with the lowest amount of genetic drag on LG12 (4%, Table 1).

While estimating the percentage of genetic drag associated with *Fb_E*, we also verified the pedigrees of the seedlings. This allowed us to discover a mistake from 2012, which occurred while raising BC’3_5. In fact, SSR marker analyses did not confirm ‘Granny Smith’ as the father of this seedling. The most probable father is BC’1_16 as: (i) BC’3_5 inherited all the alleles of BC’1_16 in repulsion with *Fb_E* from the top of the LG 12 to the interval between the SSR markers CH03c02 and Hi07f01, where a crossing-over occurred so that BC’3_5 inherited *Fb_E*, and (ii) three plantlets of this genotype flowered in 2012 in the greenhouse where the experiment was conducted (Table 1). How this outcrossing occurred is unclear. The quarantine greenhouse prevents the entry of the classical apple pollinator insects, like bees (wild bees included), bumblebees, hoverflies, and owlet moths (Jacquemart 2007; Reim 2008). The only insects sporadically present in the cabin were aphids and trips, but insecticides against them were applied and both insect species are not known to be apple pollinators. Kato and Soejima (2001) and Soejima (2007) studied the pollination success (pollinations that finally led to apples with seeds) in absence of insects acting as vectors but in presence of wind. Under these conditions, apples were produced at a radius of about 1 m from the pollinator. Wind is obviously absent in the greenhouse cabin, but a flow of air due to air conditioning is present. Whether this is sufficient to lead to undesired pollination is unclear but judged as improbable. Two hypotheses remain: (i) accidental contact of the flowers of the two genotypes due to the relatively high density of the plants in the cabin, or (ii) residues of pollen from BC’1_16 flowers on the paintbrush that was used to pollinate BC’2_32. Between two different pollinations, we cleaned the paintbrushes with 70% ethanol. It is plausible that this cleaning step was mistakenly omitted or was not sufficient to remove all BC’1_16 pollen. To reduce the risk of outcrossing, the anthers of the flowers that are chosen for pollination could be removed or mono-use paintbrushes or similar tools could be used.

Three BC’3_2014/BC’4 seedlings (BC’3_2014_10, BC’4_10 and 11, Table 1) showed the habitus we named “ananas”. All three seedlings have the same father, i.e., Modi^®^ in common, and two of them also share the same mother (BC’3_5). The cause of this particular habitus is unknown. It was not observed in earlier generations; however, it cannot be excluded that it may have been masked by the presence of *BpMADS4* or that such seedlings have been discarded at an early stage as judged unsuitable for breeding. Seedling BC’3_2014_10 particularly shows a habitus very similar to transgenic apple plantlets with impaired phloretin production (Dare et al. 2013, 2017). These later plantlets also have very short internodes and lanceolate leaves. Whether a reduced phloretin production is the cause of the ‘ananas’ habitus observed in these three seedlings will need further studies.

### Fire blight resistance maintained across the generations

The level of fire blight resistance of four groups of genotypes which were subdivided according to the inherited combination of *BpMADS4 and Fb_E* was previously studied by Le Roux et al. (2012) using F1 genotypes derived from the cross T1190 × ‘Evereste’. They observed a reduction of the average resistance of the genotypes carrying *BpMADS4* and *Fb_E* compared to the group of genotypes carrying only *Fb_E*, and hypothesized that the slender habitus of the *BpMADS4* genotypes may favor the invasion of shoots by bacteria. We did not observe this reduction of resistance in the BC’3 seedlings we tested (Fig. 3). These two groups of genotypes did not show a statistically significant difference (*P *< 0.05) with regard to fire blight resistance and were not statistically different from ‘Evereste’, too. On the contrary, we observed a statistically significant difference between the group of genotypes without *BpMADS4* and without *Fb_E* and the group carrying only *BpMADS4*. The latter group was more resistant than the former. This increase of resistance could be due to a favorable combination of unknown QTL for fire blight resistance or to chance, as all groups are composed of a relatively low number of genotypes. The phenotypic assessment of fire blight resistance of genotypes carrying *BpMADS4* is challenging, because the shoots of these genotypes may already stop growing and produce a terminal flower after a few weeks of cultivation. If this happens before the material is inoculated, the resistance level of these genotypes may be overestimated, as shoots that are no longer actively growing generally show increased resistance. Due to this, if molecular markers tightly linked to the fire blight resistance locus are available, it is recommended to identify the genotypes carrying *BpMADS4* and the resistance locus using these markers. Verifying maintenance of the effect of the resistance locus after few crosses could probably be phenotypically assessed more reliably in the genotypes that according to the molecular marker analysis are carrying the resistance locus but lacking *BpMADS4*.

## Conclusions

Applying the above-mentioned suggestions, advanced selections with genes of “wild” origin, purified from genetic drag, can be developed 4–5 times faster than by classical breeding. USDA regulators have decided that null segregants (e.g., the final products of the early flowering system) will be outside regulatory authority, as long as these genotypes have been tested for phenotype and molecularly shown to not contain transgenes or pieces of transgenes (USDA 2011, 2014; McGarry et al. 2017). As a consequence of this decision, the early flowering system based on the line T1190 is currently in use by the USDA-NIFA-SCRI RosBREED project to speed up the pyramiding of resistance loci for fire blight and apple scab resistance in apple (Norelli et al. 2015). Large segregating populations are obtained using early flowering transgenic genotypes as male parents (pollen donors) and non-transgenic trees in the orchard as female parents. Containment of transgenic pollen and seeds is achieved by netting (JL Norelli, pers. communication).

The definition of the legal status of the final products (i.e., the null segregants) in Europe is still pending. As long as this is the case, European breeders cannot decide if it is worth investing in this approach which makes use of a genetic modification to break juvenility, but absent in the final product.

### Author contribution statement

IS and JM performed the work, HF and M-VH provided the line T1190 used in this study, GB supported the phenotypic assessment of the fire blight resistance level of the plant material, AP promoted and supervised the work. All the authors actively participated to the writing of the MS.

## Electronic supplementary material

Below is the link to the electronic supplementary material.

Supplementary material 1 (DOCX 32 kb)
