# Supplementary material for: Generation of advanced fire blight-resistant apple (Malus × domestica) selections of the fifth generation within 7 years of applying the early flowering approach
Source: Planta. 2018 Mar 14;247(6):1475–88. doi: 10.1007/s00425-018-2876-z (PMC5945749; doi:10.1007/s00425-018-2876-z)
Supplement: Supplementary file 1 — Supplementary material 1 (DOCX 32 kb) [file 425_2018_2876_MOESM1_ESM.docx]

**Suppl. Table S1** Statistics of the introgression of *Fb_E* in advanced apple selections. Genotypes indicated produced apples with seeds (exceptions: Maloni Sally® pollinated with T×E_F1_81 for completeness with regards to Le Roux et al. 2012; BC’1_16 as this seedling was among those with the least percentage of ‘Evereste’ genome, according to Le Roux et al. 2012)

| Year | Generation | Cross | N° of mother trees pollinated | N° of weeks from sowing to first flowers of GM seedling | N° of pollinated flowers | N° of harvested fruits | N° of seeds | N° of seedlings | N° of seedlings per group | | | |
| --- | --- | --- | --- | --- | --- | --- | --- | --- | --- | --- | --- | --- |
|  |  |  |  |  |  |  |  |  | *BpMADS4* | Fb_E | Fb_E + *BpMADS4* | none of both |
| 2009 | F1 | T1190 × 'Evereste' | 15 | n.a | 54 | 28 | 134 | 56 | 17 | 12 | 18 | 9 |
|  |  | 'Evereste' × T1190 | 3 | n.a | 58 | 3 | 6 | 6 | 2 | 1 | 1 | 2 |
|  |  | Total 2009 | 18 |  | 112 | 31 | 140 | 62 | 19 | 13 | 19 | 11 |
| 2010 | BC'1 | 'Topaz' × T×E_F1_74 | 1 | 15 | 62 | 5 | 16 | 10 | 3 | 4 | 3 | 0 |
|  |  | 'Topaz' × T×E_F1_81 | 1 | 15 | 67 | 8 | 49 | 35 | 9 | 11 | 11 | 4 |
|  |  | Maloni Sally^®^ × T×E_F1_74 | 1 | 15 | 50 | 7 | 33 | 26 | 9 | 7 | 2 | 8 |
|  |  | Maloni Sally^®^ × T×E_F1_81 | 2 | 15 | 135 | 0 | 0 | 0 | 0 | 0 | 0 | 0 |
|  |  | E×T_F1_5 × 'Milwa' | 1 | 44 | 30 | 9 | 39 | 19 | 3 | 5 | 9 | 2 |
|  |  | Total 2010 | 6 |  | 344 | 29 | 137 | 90 | 24 | 27 | 25 | 14 |
| 2011 | BC'2 | BC'1_7 × 'Royal Gala' | 1 | 22 | ≤ 5 | 5 | 32 | 30 | 8 | 6 | 4 | 12 |
|  |  | BC'1_16 × 'Royal Gala' | 1 | 22 | ≤ 5 | 0 | 0 | 0 | 0 | 0 | 0 | 0 |
|  |  | BC'1_19 × 'Royal Gala' | 1 | 21 | ≤ 5 | 2 | 18 | 16 | 3 | 8 | 2 | 3 |
|  |  | BC'1_21 × 'Royal Gala' | 1 | 25 | ≤ 5 | 1 | 4 | 2 | 0 | 1 | 1 | 0 |
|  |  | BC'1_74 × 'Royal Gala' | 1 | 14 | ≤ 5 | 1 | 6 | 5 | 3 | 1 | 0 | 1 |
|  |  | BC'1_75 × 'Royal Gala' | 1 | 15 | ≤ 5 | 1 | 4 | 2 | 1 | 0 | 1 | 0 |
|  |  | BC'1_81 × 'Royal Gala' | 1 | 14 | ≤ 5 | 2 | 7 | 7 | 3 | 1 | 1 | 2 |
|  |  | BC'1_85 × 'Royal Gala' | 1 | 18 | ≤ 5 | 1 | 7 | 6 | 0 | 3 | 3 | 0 |
|  |  | BC'1_96 × 'Royal Gala' | 1 | 18 | ≤ 5 | 2 | 4 | 4 | 0 | 2 | 1 | 1 |
|  |  | Total 2011 | 9 |  | >27^5^ | 15 | 82 | 72 | 18 | 22 | 13 | 19 |
| 2012 | BC'2_2012 | BC'1_16 x 'Royal Gala' | 1 | n.a^1^ | 11 | 11 | 69 | 60 | 19 | 15 | 13 | 13 |
|  |  | BC'1_16 x 'Granny Smith' | 2 | n.a^1^ | 8 | 7 | 53 | 21 | 4 | 7 | 4 | 6 |
|  |  | Total 2012 | 3 |  | 19 | 18 | 122 | 81 | 23 | 22 | 17 | 19 |
|  | BC'3 | BC'2_2 x 'Granny Smith' | 1 | 28 | 7 | 4 | 28 | 27 | 3 | 9 | 9 | 6 |
|  |  | BC'2_19 x 'Granny Smith' | 1 | 24 | 16 | 3 | 5 | 5 | 0 | 2 | 3 | 0 |
|  |  | BC'2_20 x 'Granny Smith' | 1 | 24 | 10 | 6 | 3 | 2 | 0 | 0 | 2 | 0 |
|  |  | BC'2_21 x 'Granny Smith' | 1 | 20 | 7 | 1 | 3 | 0 | 0 | 0 | 0 | 0 |
|  |  | BC'2_32 x 'Granny Smith' | 1 | 19 | 15 | 10 | 40 | 31 | 10 | 7 | 6 | 8 |
|  |  | BC'2_45 x 'Granny Smith' | 1 | 23 | 10 | 3 | 8 | 3 | 0 | 0 | 3 | 0 |
|  |  | BC'2_48 x 'Granny Smith' | 1 | 20 | 12 | 1 | 2 | 1 | 1 | 0 | 0 | 0 |
|  |  | BC'2_59 x 'Granny Smith' | 1 | 24 | 6 | 2 | 1 | 1 | 0 | 1 | 0 | 0 |
|  |  | BC'2_62 x 'Granny Smith' | 1 | 21 | 12 | 7 | 0 | 0 | 0 | 0 | 0 | 0 |
|  |  | BC'2_63 x 'Granny Smith' | 1 | 24 | 15 | 4 | 0 | 0 | 0 | 0 | 0 | 0 |
|  |  | BC'2_64 x 'Granny Smith' | 1 | 25 | 16 | 1 | 0 | 0 | 0 | 0 | 0 | 0 |
|  |  | BC'2_65 x 'Granny Smith' | 1 | 23 | 8 | 3 | 21 | 5 | 1 | 2 | 0 | 2 |
|  |  | Total 2012 | 12 |  | 134 | 45 | 111 | 75 | 16 | 21 | 23 | 17 |
| 2014 | BC'3_2014 | BC'2_16_2012 x Modi^®^ | 1 | 23^2^ | 14 | 1 | 4 | 0 | 0 | 0 | 0 | 0 |
|  |  | BC'2_16_2012 x Kanzi^®^ |  |  |  | 2 | 8 | 6 | 0 | 2 | 0 | 4 |
|  |  | BC'2_22_2012 x Modi^®^ | 1 | 20^2^ | 11 | 2 | 13 | 10 | 2 | 2 | 4 | 2 |
|  |  | BC'2_43_2012 x 'Fuji' | 1 | 59^3^ | 24 | 4 | 12 | 12 | 2 | 2 | 5 | 3 |
|  |  | BC'2_43_2012 x Modi^®^ |  |  |  | 1 | 4 | - | - | - | - | - |
|  |  | BC'2_43_2012 x 'Ladina' |  |  |  | 2 | 23 | 22 | 4 | 6 | 6 | 6 |
|  |  | BC'2_43_2012 x Kanzi^®^ |  |  |  | 5 | 44 | - | - | - | - | - |
|  |  | BC'2_82_2012 x Modi^®^ | 1 | 27^2^ | 19 | 2 | 10 | - | - | - | - | - |
|  |  | Total 2014 | 4 |  | 68 | 19 | 118 | 50 | 8 | 12 | 15 | 15 |
|  | BC'4 | BC'3_5 x Modi^®^ | 1 | 24^2^ | 7 | 3 | 13 | 11 | 4 | 5 | 1 | 1 |
|  |  | BC'3_21 x 'Ladina' | 1 | 22^2^ | 38 | 1 | 2 | 2 | 0 | 0 | 1 | 1 |
|  |  | BC'3_21 x Kanzi^®^ |  |  |  | 1 | 2 | 2 | 0 | 0 | 1 | 1 |
|  |  | BC'3_33 | 1 | 59^3^ | 2 | 0 | 0 | 0 | 0 | 0 | 0 | 0 |
|  |  | BC'3_37 x ' Ladina' | 1 | 26^2^ | 27 | 2 | 9 | 8 | 1 | 3 | 2 | 2 |
|  |  | BC'3_39 x Modi^®^ | 1 | 30^2^ | 8 | 3 | 9 | 9 | 1 | 3 | 3 | 2 |
|  |  | BC'3_41 x Modi^®^ | 1 | 24^2^ | 23 | 1 | 4 | 2 | 0 | 0 | 0 | 2 |
|  |  | BC'3_46 x Kanzi^®^ | 1 | 59^3^ | 16 | 6 | 47 | 19^4^ | 4 | 4 | 9 | 2 |
|  |  | BC'3_49 x Kanzi^®^ | 1 | 30^2^ | 8 | 3 | 8 | 4 | 1 | 1 | 1 | 1 |
|  |  | BC'3_50 x 'Fuji' | 1 | 25^2^ | 22 | 1 | 6 | 5 | 2 | 1 | 1 | 1 |
|  |  | BC'3_50 x Modi^®^ |  |  |  | 3 | 13 | 12 | 3 | 3 | 4 | 2 |
|  |  | BC'3_60 x Modi^®^ | 1 | 22^2^ | 13 | 2 | 11 | 6 | 0 | 1 | 1 | 4 |
|  |  | BC'3_66 | 1 | 8^2^ | 15 | 0 | 0 | 0 | 0 | 0 | 0 | 0 |
|  |  | BC'3_68 x Kanzi^®^ | 1 | 23 | 43 | 8 | 44 | 20^4^ | 4 | 5 | 8 | 3 |
|  |  | BC'3_69 x Modi^®^ | 1 | 8^2^ | 20 | 4 | 8 | 7 | 3 | 0 | 4 | 0 |
|  |  | BC'3_69 x 'Kanzi' |  |  |  | 1 | 3 | 2 | 0 | 0 | 1 | 1 |
|  |  | BC'3_71 x 'Fuji' | 1 | 24^2^ | 36 | 3 | 4 | 4 | 0 | 2 | 1 | 1 |
|  |  | BC'3_75 | 1 | 29^2^ | 33 | 0 | 0 | 0 | 0 | 0 | 0 | 0 |
|  |  | Total 2014 | 15 |  | 311 | 42 | 183 | 113 | 23 | 28 | 38 | 24 |
| Grand Total  per generation | F1 |  | 18 | 18^6^ | 112 | 31 | 140 | 62 | 19 | 13 | 19 | 11 |
|  | BC'1 |  | 6 | 19^6^ | 344 | 29 | 137 | 90 | 24 | 27 | 25 | 14 |
|  | BC'2 |  | 12 | 25^6,7^ | >46 | 33 | 204 | 153 | 41 | 44 | 30 | 38 |
|  | BC'3 |  | 16 | 27^6,7^ | 202 | 64 | 229 | 125 | 24 | 33 | 38 | 32 |
|  | BC'4 |  | 15 | - | 311 | 42 | 183 | 113 | 23 | 28 | 38 | 24 |

| ^1^ grafted plants, see 2011 data. |
| --- |
| ^2^ data from 2013 |
| ^3^ seedling did not flower in 2013 |
| ^4^ 20 out of all available seeds have been sown |
| ^5^ See Le Roux et al. 2012  ^6^ Average number of weeks needed to produce flowers per generation  ^7^ Excluding the genotypes that did not flower in 2013, the average time needed to flower for the first time decreased to 23 weeks for both BC’2 and ‘3 generations |
